# The effect of Timolol 0.5% on the correction of myopic regression after LASIK

**DOI:** 10.1097/MD.0000000000006782

**Published:** 2017-04-28

**Authors:** Hong Qi, Caifeng Gao, Yaxin Li, Xue Feng, Miao Wang, Yu Zhang, Yueguo Chen

**Affiliations:** a Department of Ophthalmology, Peking University Third Hospital; b Key Laboratory of Vision Loss and Restoration, Ministry of Education; c Moslem Hospital, Beijing, China.

**Keywords:** LASIK, myopic, Timolol

## Abstract

**Backgroud:**

Postlaser in situ keratomileusis (post-LASIK) refractive regression is defined as the gradual, partial, or total loss of initial correction that limits the predictability, efficiency, and long-term stability of LASIK. Our study assesses the effect of Timolol 0.5% on the correction of myopic regression after LASIK.

**Methods:**

This prospective, randomized, controlled study included 62 eyes of 62 patients with myopic regression of −1.18 ± 0.86 diopters (D) after myopic LASIK. They were randomly assigned into either Group 1 who received Timolol 0.5% eye drops for 3 months or Group 2 who received artificial tears as control (during treatment). Patients were followed an additional 2 months after cessation of eye drops treatment (posttreatment).

**Results:**

During treatment in Group 1, as the mean true intraocular pressure (IOPT) lowered significantly, regression stopped. As the mean IOPT increased significantly posttreatment and returned to its pretreatment level, regression recurred. The effective rate of Timolol therapy dropped from 62.5% during treatment to 40.6% posttreatment. On the contrary in Group 2, although the mean IOPT did not change significantly, regression continually happened as time passed. During treatment, the mean IOPT, uncorrected visual acuity, spherical equivalent (SE), and corneal refractive power showed significant difference between the 2 groups. In Group 1, the differences of effective rate of Timolol therapy between each of the 2 subgroups of age, gender, preoperative SE (PSE), or pretreatment time (how long we start treatment with Timolol post-LASIK) were not statistically significant.

**Conclusion:**

IOP-lowering eye drop Timolol was effective for the correction of myopic regression when a 0.5-D or greater myopic shift is detected after LASIK in patients regardless of age, gender, PSE, or anytime we started the treatment only if regression happened. However, the myopic regression recurred after cessation of Timolol treatment.

## 1 Introduction

Postlaser in situ keratomileusis (post-LASIK) refractive regression is defined as the gradual, partial, or total loss of initial correction that limits the predictability, efficiency, and long-term stability of LASIK. LASIK was improved in the past few years, but myopic regression was inevitable. Chen et al^[1]^ reported that the rate of myopic regression was 21% in average-risk eyes. Although the exact mechanism of refractive regression after LASIK is unclear, previous studies have suggested a forward shift of the cornea as 1 possible explanation of myopic regression.^[2–4]^ Currently, the treatment for visually significant myopic regression after LASIK was repeat LASIK that includes lifting the flap, cutting a new flap, or surface ablation.^[5]^ The 3 procedures all have potential risks such as epithelial ingrowth, and so on.^[5]^

Our 2006 study^[4]^ indicated that lowering the intraocular pressure (IOP) preoperatively and postoperatively may be an effective way to prevent myopic regression and iatrogenic keratectasia after LASIK. Studies have reported a relationship between the elevation of IOP and corneal protrusion.^[2,6,7]^ Increasing attention is now being paid to nonsurgical approaches to treat small amounts of myopic regression. In 2008, Kamiya et al^[8]^ demonstrated that topical application of an IOP-lowering eye drop was effective for the correction of myopic regression that presumably resulted from the backward movement of the cornea and the flattening of its curvature after LASIK. Although the refractive effect of this treatment is mild (approximately 0.5 diopters [D]), it has advantages over enhancement ablation because it seems to be less invasive and to cause fewer side effects (e.g., keratectasia) in light of the biomechanical stability of the cornea. Many questions still remain, however, regarding the topical application of IOP-lowering drops to treat myopic regression after LASIK.^[5]^

Timolol (Wuhan Pharmaceutical Limited-liability Company, Wuchang, Wuhan, China) 0.5% eye drops, a nonselective B-blocker, are used most commonly in China in patients with glaucoma. Timolol reduces IOP by decreasing aqueous humor production. As IOP-lowering eye drops have been proven to be effective for the correction of myopic regression after LASIK,^[8]^ our study considered 2 issues: what happens to the myopic regression when the application of the IOP-lowering eye drops is stopped? and what factors influence the effectiveness of IOP-lowering eye drops for the correction of myopic regression after LASIK?

## 2 Patients and methods

### 2.1 Continuous variables were expressed as mean ± standard deviation

Sixty-two eyes of 62 patients (30 males and 32 females) with myopic regression of −1.18 ± 0.86 D (range −0.5 to −6.63 D) after myopic LASIK from May 2012 to May 2014 were included in this prospective, randomized, controlled study. Inclusion criteria before and after LASIK were age of patients 19 years or more, patients not wearing contact lens 4 weeks before the surgery, eyes with an IOP ranging from 10 to 20 mm Hg measured by a Goldmann applanation tonometer (YZ30; Suzhou Medical Instrument General Factory, Suzhou, China) and absence of a glaucoma, eyes with normal peripheral retina, and eyes with stable refractive error at least 12 months before surgery. Exclusion criteria before and after LASIK were presence of keratoconus or keratoconus suspect determined on the basis of the corneal topography using a WaveLight Allegro Topolyzer (Lumenis, Dreieich, Germany), eyes with central islands, eyes with active inflammation, eyes with Schirmer test less than 5.0 mm, eyes with a minimum residual untreated posterior corneal thickness of 250 μm, and patients with history of ocular trauma. All patients had bilateral LASIK. In order to obviate intereye correlation, only 1 eye of a patient was included.

Informed consents were obtained from all patients after explaining the nature and possible outcomes of the study. The study protocol was conducted in accordance with the ethical principles outlined in the Declaration of Helsinki. The study followed the guidelines required by the Institutional Review Board and Ethics Committee of Peking University Third Hospital.

LASIK was performed using an excimer laser system (Allegretto Eye-Q; WaveLight, Erlangen, Germany). An automated M2 90 microkeratome (Moria, Antony, France) was used to create a 120- to 150-μm-thick superior hinge flap measuring 9 mm in diameter. The ablation zone diameter was 6.0 or 6.5 mm. In all eyes, the preoperative manifest refraction was selected as the target myopic correction.

Patients were examined regularly at the preoperative and postoperative visits (first day, first week, 1, 3, 6, 12, and 24 months after LASIK). Patients were also examined timely when complains of decreased vision acuity occurred. Myopic regression was defined as myopic shift of 0.5 D or greater in manifest refraction from the first postoperative month after LASIK.

A total of 62 eyes were randomly assigned into either Group 1 (32 eyes) or Group 2 (30 eyes). Group 1 (32 eyes) included patients who received Timolol 0.5% eye drops twice daily for 3 months; Group 2 (30 eyes) included patients who received artificial tears (Systane Ultra; Alcon, Fort Worth, Texas) in the same manner as that in Group 1. The administration of eye drops was started 7.9 ± 6.8 months (range 1–21 months) after LASIK. The patients were followed for an additional 2 months after cessation of eye drops.

Before treatment with eye drops (pretreatment), after the treatment with eye drops for 3 months (during treatment), and 2 months after cessation of eye drops (posttreatment), we performed the following examinations: logarithm of the minimum angle of resolution (logMAR) of uncorrected visual acuity (UCVA), logMAR of best spectacle-corrected visual acuity (BSCVA), spherical equivalent (SE), and IOP with a Goldmann applanation tonometer. The corneal refractive power (RP) and central corneal thickness (CCT) were obtained with the Pentacam system (Oculus Optikgeräte GmbH, Wetzlar, Germany). We used the correction algorithms^[9]^ to estimate the true IOP after LASIK (Kohlhaas, IOPT = IOPG + 23.28 − 0.0423 CCT; IOPT = true intraocular pressure [mm Hg], IOPG = Goldmann applanation tonometric IOP [mm Hg]).

We used one-way Analysis of Variance (ANOVA) test to compare the values within groups in different times and independent samples *t* test to compare the results between the 2 groups. In Group 1, we analyzed preoperative and postoperative variables including age and gender of the patients, the preoperative SE (PSE) of the myopic regressive eye, and how long we start treatment with Timolol post-LASIK (pretreatment time). Chi-square test was used to analyze the effective rate of Timolol therapy of these variables. A *P* value ≤.05 was considered statistically significant. All statistical analyses were performed using SPSS 16.0 for Windows (SPSS, Chicago, IL).

## 3 Results

As shown in Table 1, no significant differences with respect to patient age, PSE, preoperative CCT, and laser ablation depth were found between the 2 groups (all *P* > .05, independent samples *t* test). In each of the 2 groups, the logMAR BSCVA in all eyes were above 0, and there were no significant differences among the 3 time points (pre-, during, and posttreatment) (*P* > .05, one-way ANOVA test). No serious Timolol-related ocular surface diseases or cardiovascular and respiratory side effects were found in all patients.

**Table 1:**
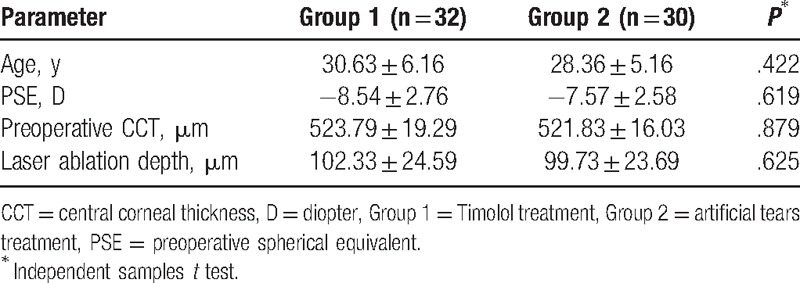
Preoperative data in eyes with myopic regression after LASIK in the 2 treatment groups.

**Figure 1:**
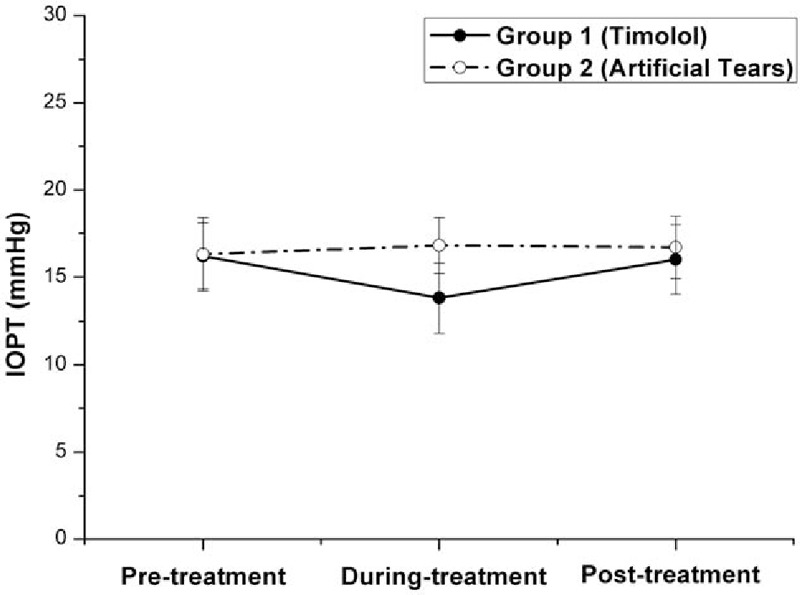
Graph demonstrating the true intraocular pressure (IOPT) in eyes with myopic regression after laser in situ keratomileusis in the 2 treatment groups in different follow-up visits. Before treatment with eye drops (pretreatment), the difference in the mean IOPT was not statistically significant between the 2 groups (*P* = .715, independent samples *t* test). Three months after the twice-daily application of Timolol (during treatment), the mean IOPT gained 2.4-mm Hg (14.8%) reduction from the baseline (pretreatment) in Group 1 (from 16.2 pretreatment to 13.8 mm Hg during treatment, *P* = .000, n = 32, one-way ANOVA test) and gained 3.0 mm Hg lower than the mean IOPT in Group 2 (*P* = .000, independent samples *t* test). Two months after cessation of Timolol treatment (posttreatment), the mean IOPT in Group 1 increased significantly (from 13.80 mm Hg during treatment to 16.0 mm Hg posttreatment, *P* = .000, n = 32, one-way ANOVA test) and returned to its own pretreatment level (16.2 mm Hg, *P* = .711, n = 32, one-way ANOVA test). The difference of the mean IOPT posttreatment between the 2 groups was not statistically significant (*P* = .092, independent samples *t* test). ANOVA = analysis of variance.

**Figure 2:**
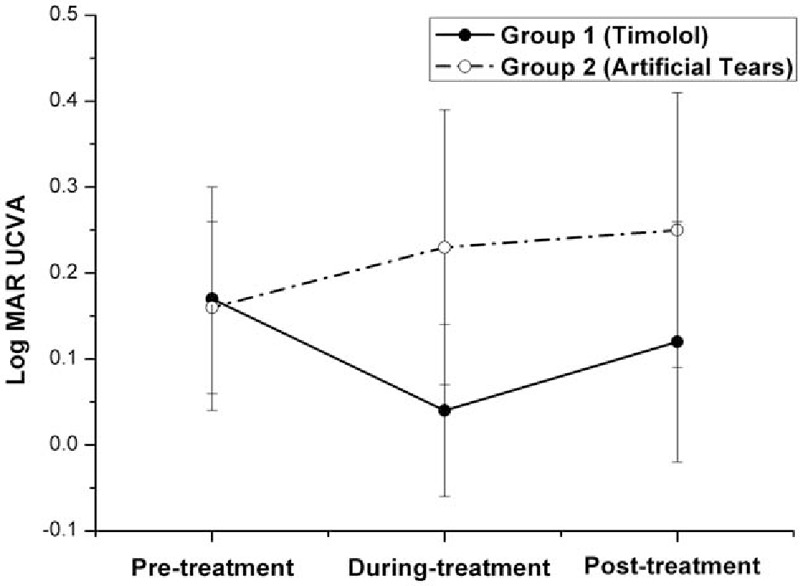
Graph demonstrating the logarithm of the minimum angle of resolution (logMAR) uncorrected visual acuity (UCVA) in eyes with myopic regression after laser in situ keratomileusis in the 2 treatment groups in different follow-up visits. Before treatment with eye drops (pretreatment), the difference in the mean logMAR UCVA was not statistically significant between the 2 groups (*P* = .667, independent samples *t* test). Three months after the twice-daily application of Timolol (during treatment), the mean logMAR UCVA decreased significantly in Group 1 (from 0.17 pretreatment to 0.04 during treatment, *P* = .000, n = 32, one-way ANOVA test) and gained 0.19 lower than the mean logMAR UCVA in Group 2 (*P* = .000, independent samples *t* test). Two months after cessation of Timolol treatment (posttreatment), the mean logMAR UCVA in Group 1 increased significantly (from 0.04 during treatment to 0.12 posttreatment, *P* = .016, n = 32, one-way ANOVA test) and almost returned to its own pretreatment level (0.17, *P* = .122, n = 32, one-way ANOVA test). But it still was significantly lower than the mean logMAR UCVA in Group 2 (*P* = .002, independent samples *t* test). ANOVA = analysis of variance, logMAR = logarithm of the minimum angle of resolution.

**Figure 3:**
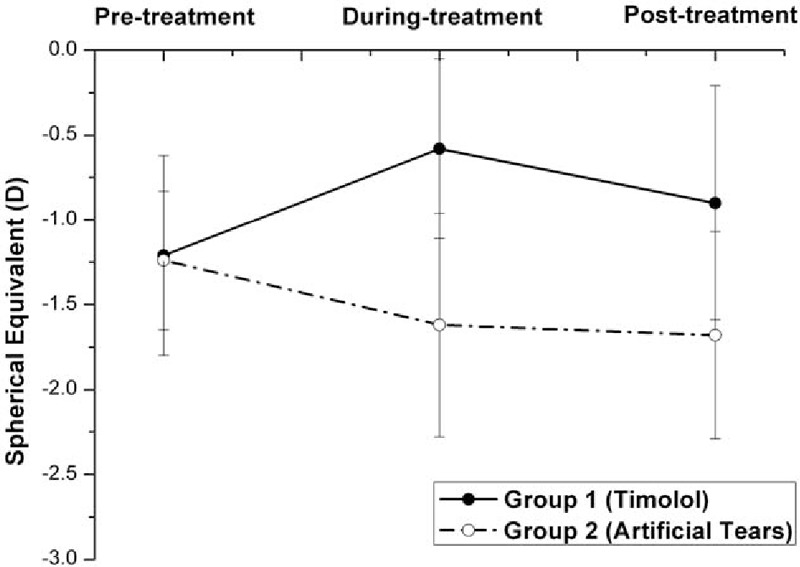
Graph demonstrating the spherical equivalent (SE) in eyes with myopic regression after laser in situ keratomileusis in the 2 treatment groups in different follow-up visits. Before treatment with eye drops (pretreatment), the difference in the mean SE was not statistically significant between the 2 groups (*P* = .442, independent samples *t* test). Three months after the twice-daily application of Timolol (during treatment), the mean SE increased significantly in Group 1 (from −1.21 D pretreatment to −0.58 D during treatment, *P* = .000, n = 32, one-way ANOVA test) and gained 1.04 D higher than the mean SE in Group 2 (*P* = .000, independent samples *t* test). Two months after cessation of Timolol treatment (posttreatment), the mean SE in Group 1 decreased significantly (from −0.58 D during treatment to −0.90 D posttreatment, *P* = .043, n = 32, one-way ANOVA test), but it still significantly was higher than its own pretreatment level (−1.21, *P* = .038, n = 32, one-way ANOVA test) and than the mean SE posttreatment in Group 2 (*P* = .000, independent samples *t* test). ANOVA = analysis of variance.

**Figure 4:**
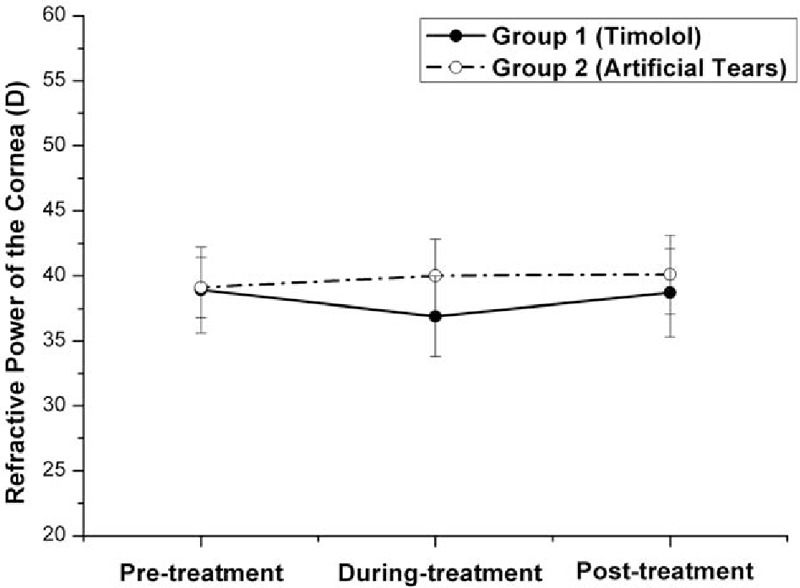
Graph demonstrating the refractive power (RP) of the cornea in eyes with myopic regression after laser in situ keratomileusis in the 2 treatment groups in different follow-up visits. Before treatment with eye drops (pretreatment), the difference in the mean RP was not statistically significant between the 2 groups (*P* = .913, independent samples *t* test). Three months after the twice-daily application of Timolol (during treatment), the mean RP decreased significantly in Group 1 (from 38.9 D pretreatment to 36.9 D during treatment, *P* = .018, n = 32, one-way ANOVA test) and gained 3.1 D lowered than the mean RP in Group 2 (*P* = .000, independent samples *t* test). Two months after cessation of Timolol treatment (posttreatment), the mean RP in Group 1 increased significantly (from 36.9 D during treatment to 38.7 D posttreatment, *P* = .036, n = 32, one-way ANOVA test) and returned to its own pretreatment level (38.9 D, *P* = .777, n = 32, one-way ANOVA test). The difference of the mean RP posttreatment between the 2 groups was not statistically significant (*P* = .069, independent samples *t* test). ANOVA = analysis of variance.

As shown in Table 2 and Figs. 1–4, the differences in the mean IOPT, logMAR UCVA, SE, and RP before treatment with eye drops (pretreatment) were not statistically significant between the 2 groups (all *P* > .05, independent samples *t* test).

**Table 2:**
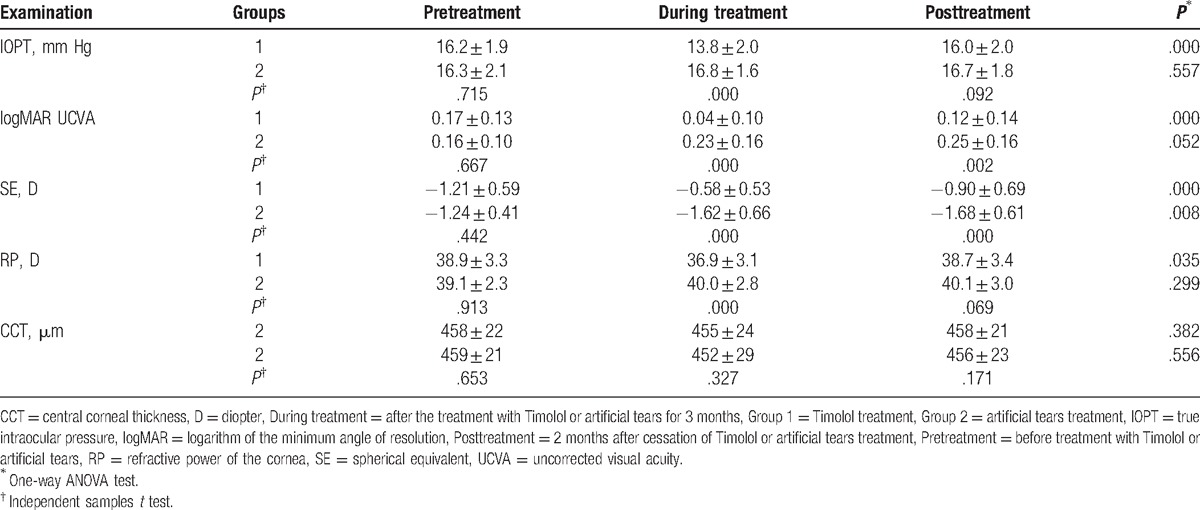
Comparison of data in eyes with myopic regression after LASIK in the 2 treatment groups in different follow-up visits.

**Figure 5:**
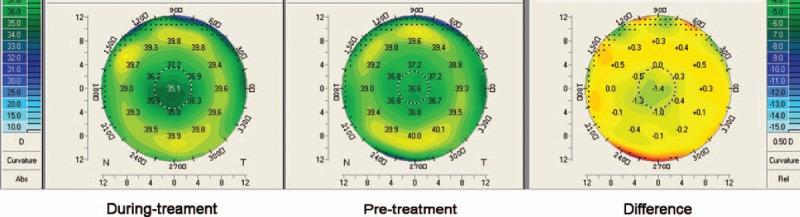
Map (Pentacam) showing the difference of corneal refractive power (RP) of an example eye in Group 1. Three months after the twice-daily application of Timolol (during treatment), the RP in the central cornea decreased (left), compared to its pretreatment level (center), 1.4 D difference of RP was observed in the central cornea (right).

As shown in Table 2 and Figs. 1–5, the mean IOPT, logMAR UCVA, SE, and RP were significantly different among the 3 time points in Group 1 (*P* < .05, n = 32, one-way ANOVA test). As the mean IOPT ± standard deviation (SD) lowered significantly from 16.2 ± 1.9 mm Hg pretreatment to 13.8 ± 2.0 mm Hg during treatment (*P* = .000), the mean logMAR UCVA ± SD was significantly improved from 0.17 ± 0.13 pretreatment to 0.04 ± 0.10 during treatment (*P* = .000). The mean SE ± SD improved significantly from −1.21 ± 0.59 to −0.58 ± 0.53 D (*P* = .000). The mean RP ± SD decreased significantly from 38.9 ± 3.3 to 36.9 ± 3.1 D (*P* = .018). As the mean IOPT ± SD increased significantly from 13.8 ± 2.0 mm Hg during treatment to 16.0 ± 2.0 mm Hg posttreatment (*P* = .000), accordingly, the mean logMAR UCVA ± SD increased significantly from 0.04 ± 0.10 to 0.12 ± 0.14 (*P* = .016), the mean SE ± SD regressed significantly from −0.58 ± 0.53 to −0.90 ± 0.69 D (*P* = .043), and the mean RP ± SD increased significantly from 36.9 ± 3.1 to 38.7 ± 3.4 D (*P* = .036). Two months after cessation of Timolol treatment (posttreatment), IOPT ± SD, logMAR UCVA ± SD, and RP ± SD almost returned to their pretreatment levels (*P* > .05). But SE ± SD still showed minor improvement, from −1.21 ± 0.59 pretreatment to −0.90 ± 0.69 posttreatment (*P* = .038).

On the contrary, although the mean IOPT ± SD did not change significantly among the 3 time points in Group 2 (*P* = .557, n = 30, one-way ANOVA test), regression happened as time passed with the mean SE decreased significantly (*P* = .008), and the mean logMAR UCVA and RP increased (although *P* > .05) (Table 2 and Figs. 1–4).

As shown in Table 2 and Figs. 1–4, after the treatment with eye drops for 3 months (during treatment), the mean IOPT, logMAR UCVA, SE, and RP showed significant difference between the 2 groups (all *P* = .000, independent samples *t* test). Even 2 months after cessation of eye drops (posttreatment), with the mean IOPT and RP lower in Group 1 than in Group 2 (although *P* > .05), the mean logMAR UCVA and SE still showed significant improvement in Group 1 than in Group 2 (*P* < .05).

**Figure 6:**
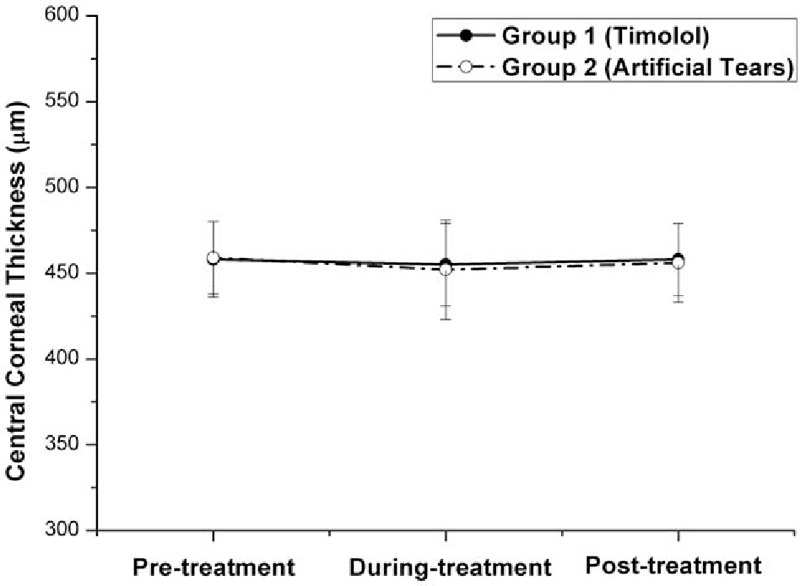
Graph demonstrating the central corneal thickness (CCT) in eyes with myopic regression after laser in situ keratomileusis in the 2 treatment groups in different follow-up visits. Before treatment with eye drops (pretreatment), the differences in the mean CCT were not statistically significant between the 2 groups (*P* = .653, independent samples *t* test). The differences in the mean CCT ± SD among the 3 time points (pre-, during, and posttreatment) were not statistically different in each of the 2 groups (*P* > .05, one-way ANOVA test). ANOVA = analysis of variance.

As shown in Table 2 and Fig. 6, the differences in the mean CCT ± SD among the 3 time points (pre-, during, and posttreatment) were not statistically significant in each of the 2 groups (*P* > .05, one-way ANOVA test).

Treatment was defined as effectiveness if the SE improved more than 0.5 D. In Group 1, 20 of 32 eyes showed the effectiveness after the treatment with Timolol for 3 months (during treatment), the effective rate of Timolol therapy was 62.5%. The effective rate dropped to 40.6% (13 of 32 eyes showed the effectiveness) 2 months after cessation of eye drops (posttreatment). There were preoperative (pre-LASIK) and pretreatment (pretreatment with Timolol) variables that may have influenced the effective rate of the application of Timolol, such as age and gender of the patients, the PSE of the myopic regressive eye, and the pretreatment time. As shown in Table 3, the effective rate of Timolol therapy in the subgroup of females, age <30 years, PSE < −10 D, and pretreatment time <6 months post-LASIK, was a little higher than in the subgroup of males, age ≥30 years, PSE ≥ −10 D, and pretreatment time ≥6 months post-LASIK. But the differences between each of the 2 subgroups were not statistically significant (all *P* > .05, chi-square test).

**Table 3:**
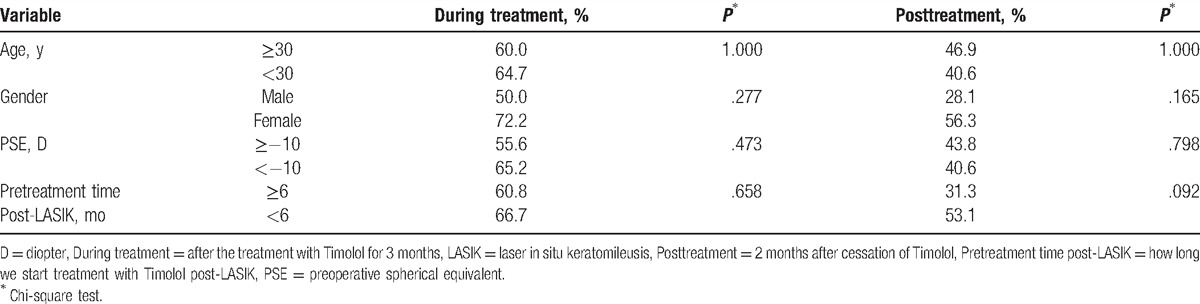
The effective rate in eyes with myopic regression after LASIK in Group 1 (Timolol treatment, n = 32) in different follow-up visits.

## 4 Discussion

### 4.1 If the IOP-lowering eye drug is effective for the correction of myopic regression after LASIK, what happens to the myopic regression when the application of the IOP-lowering eye drops is stopped?

Even now, the mechanism of refractive regression after LASIK is not fully elucidated. However, most previous studies agree that myopic regression after LASIK is attributable to epithelial hyperplasia and stromal remodeling.^[10,11]^ Pan et al^[12]^ compared regressive eyes with nonregressive eyes after LASIK and indicated that refractive regression after LASIK might be mainly induced by corneal protrusion rather than by CCT. Our study also demonstrated that there were no significant changes in CCT in each of the 2 groups (Table 2 and Fig. 6), indicating that changes in corneal thickness play a subtle role in the total refraction of the eye.

The corneal biomechanical strength is the barrier to resisting the IOP.^[4]^ If the corneal biomechanical strength against IOP is not powerful enough, the forward shift of the cornea will occur, which counteracts the refractive effects of surgery, implying that it can be one of the factors responsible for refractive instability after excimer laser surgery.^[13]^ This is the problem existing in the enhancement ablation for the correction of myopic regression after surgery. If corneal tissue is subtracted excessively from the residual cornea, structural integrity of the cornea is compromised further, resulting in greater forward shift and further myopic regression.^[13]^

In recent years, refractive surgeons have tried nonsurgical approaches to treat myopic regression after LASIK, and the IOP-lowering eye drop Nipradilol^[8]^ was proven to be effective. Our study demonstrated that IOP-lowering eye drop Timolol was effective for the correction of refractive regression. Three months after the twice-daily application of Timolol (Table 2 and Figs. 1–5) in Group 1, as the mean IOPT gained 14.8% reduction from the baseline (from 16.2 mm Hg pretreatment to 13.8 mm Hg during treatment, *P* = .000) or gained 3.0 mm Hg lowered than the mean IOPT in Group 2 (*P* = .000), the mean RP decreased significantly (2 D, from 38.9 D pretreatment to 36.9 D during treatment, *P* = .018). Accordingly, the mean logMAR UCVA decreased significantly (0.13, from 0.17 pretreatment to 0.04 during treatment, *P* = .000), and the mean SE improved significantly (0.63 D, from −1.21 D pretreatment to −0.58 D during treatment, *P* = .000). In 32 eyes, 20 eyes showed the effectiveness of Timolol treatment (effective rate 62.5%) with SE improved more than 0.5 D. Three months after the twice-daily application of Timolol, significant differences with respect to the mean RP, logMAR UCVA, and SE were found between the 2 groups (all *P* = .000).

This study explored what would happen to the myopic regression when the IOP-lowering eye drops were stopped. Two months after cessation of Timolol treatment in Group 1 (Table 2 and Figs. 1–4), the mean IOPT increased significantly (2.2 mm Hg, from 13.80 mm Hg during treatment to 16.0 mm Hg posttreatment, *P* = .000) and returned to the pretreatment level (16.2 mm Hg, *P* > .05). Regression recurred with the mean RP and logMAR UCVA increased significantly, and SE decreased significantly (all *P* < .05). So the effective rate of Timolol therapy dropped from 62.5% during treatment to 40.6% posttreatment. When compared with their pretreatment levels, except for the mean SE still showing minor improvement (0.21 D, from −1.21 D pretreatment to −0.90 D posttreatment, *P* = .038), the mean logMAR UCVA and RP almost returned to their baseline (*P* > .05).

These results show that the morphologic properties of the cornea are easily affected by subtle changes in IOP when corneal rigidity is impaired by flap manipulation and laser ablation such as LASIK. IOP reduction may have induced a backward shift of the cornea and reduction of RP, resulting in the improvement in refraction and visual acuity in post-LASIK eyes. Although the myopic regression recurred once the administration of IOP-lowering eye drops had stopped, the mean logMAR UCVA and SE still showed significant improvement in Group 1 than in the control group (*P* < .05).

### 4.2 What factors may influence the effectiveness of IOP-lowering eye drops for the correction of myopic regression after LASIK?

As shown in Table 3, age and gender of the patients did not impact the effective rate of Timolol therapy, nor did the PSE of the myopic regressive eye and the pretreatment time. In this study, we started topical administration of Timolol when a 0.5-D or greater myopic shift was detected. The pretreatment time varied from 1 to 21 months (7.9 ± 6.8 months) after LASIK. As shown in Table 3, the effective rate of Timolol therapy in the subgroup, in which we start treatment with Timolol post-LASIK within 6 months, was not significantly higher than in the subgroup that was treated for longer than 6 months (*P* > .05). Although a higher PSE is more predisposed to lead to myopic regression,^[2]^ the effective rate of Timolol therapy in the subgroup with PSE ≥ −10 D was also not significantly lower than in the subgroup with PSE < −10 D (*P* > .05). These data indicated that Timolol might be effective for the correction of myopic regression when a 0.5-D or greater myopic shift was detected after LASIK in patients regardless of age, gender, PSE, or anytime we started the treatment only if regression happened.

In 12 (37.5%) of 32 eyes, however, the twice-daily administration of Timolol was not effective for the correction of refractive regression, although there was some lowering effect of the IOP in all eyes. The reasons may be as follows:

(1) The IOPT may not be low enough for those noneffective eyes. We combined the use of the IOPT-lowering drug Brimonidine (Allergan Pharmaceuticals Ireland) (a highly selective a2 adrenergic receptor agonist) with Timolol and gained a higher effective rate (data not shown). A recent study showed that Brimonidine has a significant miotic effect on pupil size.^[14]^ The application of this agent 20 minutes before activities in dimly lit areas or at night may be recommended for photic phenomena following refractive surgery.^[15]^ We are currently conducting a study to compare the results of Timolol, Brimonidine, and the combination use of Timolol with Brimonidine for the correction of myopic regression after LASIK. Not all antiglaucoma drugs, however, may contribute to an improvement in myopic regression after keratorefractive surgery. An antiglaucoma medication subcategory, the prostaglandin F2α analogs (PGAs), may offer the opposite effect in this regard. Topical application of PGAs after refractive surgeries such as LASIK may significantly reduce the CCT, as a result of prostaglandin F2α-induced upregulation of matrix metalloproteinases and subsequent effects on the extracellular matrix of the corneal stroma.^[16]^ These effects eventually may weaken the formerly destabilized cornea and subsequently may lead to progressive myopic regression, ectasia,^[17]^ or both. But the topical application of Timolol in this study did not show any effect on reducing the CCT.
(2) Refractive effects may not depend simply on the degree of IOP reduction. A higher IOP may be one of the main causative factors for structurally compromised corneas after LASIK.^[2,6,7]^ Hence, we assumed that IOP-lowering drugs may be highly effective for regression primarily caused by corneal geometrical changes.^[4]^ But many other factors such as epithelial hyperplasia, development of new stromal collagen, and nuclear sclerosis of the lens may sometimes play significant roles in myopic regression.^[18,19]^ If biomechanical properties of the cornea are still strong or normal after LASIK, or if the corneal wound healing has been stabilized, this treatment may be less effective. Further studies are required to clarify this point.

Hiatt et al^[6]^ indicated that biomechanical remodeling of the cornea had not been completed even 10 months after the application of IOP-lowering drugs. Epithelial hyperplasia after LASIK may be a natural defense mechanism in which keratocytes respond to corneal trauma and function to reconstruct and preserve the original structure and conformation of the corneal tissue. The return to normal epithelial thickness may take months or even years, and the regulatory mechanisms have not yet been characterized.^[11]^ As a result of this corneal mechanism, even though IOP is maintained in the regular range, the corneal biomechanical strength against IOP continues to change. The counteraction between the corneal biomechanical strength and the IOP is a long-term dynamic cross-action. Because it is unclear when the biomechanical properties of the cornea have stabilized, it is also unknown how long this treatment needs to be continued. If the IOP reduction has effect on the refraction, the patients need the antiglaucoma drugs continuously.^[4]^ However, there are potential side effects in long-term use of antiglaucoma medication. Long-term use may induce changes in the tear film, cornea, and conjunctival surface and the risk of serious cardiovascular and respiratory side effects.^[20–23]^ Fortunately, all patients were younger than 45 years in patients with LASIK, and so Timolol-related systematic side effects seldom occurred in this group.

The Goldmann applanation tonometer, the most widely used method of measuring intraocular pressure, is the current gold standard. However, a number of corneal parameters can affect the accuracy of this instrument. Previous studies have proven that intraocular pressure measurements after LASIK for the correction of myopia can always be underestimated.^[9,24,25]^ Kohlhaas et al^[24]^ study indicated that CCT, corneal curvature, and corneal flap stability affect the accuracy of intraocular pressure measurements after LASIK. We used the Kohlhaas correction algorithms to estimate the true IOP after LASIK.

In conclusion, we demonstrated in this study that when we started the twice-daily administration of Timolol 0.5% at 1 to 21 months (7.9 ± 6.8 months) after LASIK for 3 months, it was effective for the correction of refractive regression of −1.18 ± 0.86 D (range −0.5 to −6.63 D). But the myopic regression recurred 2 months after cessation of the application. To improve the safety, efficacy, predictability, and stability of LASIK, the intervention of the IOP-lowering drugs, a medical alternative for modulating refractive results after surgery, may be considered. However, there are still many questions remaining regarding the therapeutic use of IOP-lowering eye drugs, and the rational administration for long-term use, the combination use, and the preventive use of IOP-lowering drugs for the correction of myopic regression after LASIK requires further investigation.
